# Risk assessment for sandwich vertebral fractures in the treatment of osteoporosis vertebral compression fractures using two methods of bone cement reinforcement

**DOI:** 10.1186/s13018-023-04006-x

**Published:** 2023-07-22

**Authors:** Youzhi An, Lili Li, Xuelin Lin, Zhen Zhang, Zhaoyun Zheng, Chengjiang Wang

**Affiliations:** 1 Spine Surgery, The Second Hospital of Liaocheng Affiliated to Shandong First Medical University, No. 306, Jiankang Street, Linqing City, Shandong China; 2 Medical Oncology, The Second Hospital of Liaocheng Affiliated to Shandong First Medical University, No. 306, Jiankang Street, Linqing City, Shandong China

**Keywords:** Vertebroplasty, Kyphoplasty, Osteoporosis, Spinal fractures, Compression fracture, Sandwich vertebra

## Abstract

**Purpose:**

Bone cement augmentation surgery includes percutaneous vertebroplasty (PVP) and percutaneous kyphoplasty (PKP). In this study, we aimed to investigate the risk of sandwich vertebral fractures in the treatment of osteoporotic vertebral compression fractures via PVP and PKP.

**Methods:**

We performed a retrospective analytical study and included 61 patients with osteoporotic vertebral compression fractures who underwent PVP and PKP at the Spinal Surgery Department of The Second Hospital of Liaocheng Affiliated with Shandong First Medical University from January 2019 to January 2022. These patients were divided into the following two groups by simple random sampling: group A (*N* = 30) underwent PVP treatment and group B (*N* = 31) underwent PKP treatment. The surgical time, fluoroscopy frequency, visual analog scale (VAS) score, amount of bone cement, the leakage rate of bone cement in intervertebral space, Cobb angle, and the incidence of fractures in both groups of sandwich vertebral were recorded after 1 year of follow-up.

**Results:**

No statistically significant difference was found in terms of surgical time, fluoroscopy frequency, and VAS score between the two groups (*P* > 0.05). However, a statistically significant difference was found in terms of the amount of bone cement, the leakage rate of bone cement intervertebral space, Cobb angle, and the incidence of vertebral body fractures in both groups (*P* < 0.05). The amount of bone cement, the leakage rate of bone cement in intervertebral space, Cobb angle, and sandwich vertebral fractures were higher in Group A than in Group B.

**Conclusions:**

When PVP and PKP were performed to treat osteoporotic vertebral compression fractures, the sandwich vertebral exhibited a risk of fracture. PVP exhibited a greater relative risk than PKP, which may be due to the relatively larger amount of bone cement, higher rate of bone cement leakage in the intervertebral space, and larger Cobb angle.

## Introduction

Osteoporosis is a common disease in elderly patients, which can lead to increased bone fragility and risk of fractures [1, 2]. Epidemiological investigations suggest that the incidence of senile vertebral compression fractures in patients aged > 70 years is approximately 20%, whereas the rate in postmenopausal women is approximately 16% [3, 4]. With the advent of an aging society, osteoporosis treatment has become an important issue [5]. Unfortunately, the medical expenses of the treatment of osteoporosis and its complications are high nationwide. The most common complication of osteoporosis is osteoporotic vertebral compression fracture, which occurs in the thoracic and lumbar vertebrae. It most commonly occurs in the thoracolumbar segment.

Osteoporotic vertebral compression fractures can lead to chronic low back pain, kyphosis, and posture restriction, which can seriously affect the quality of life and physical and mental health of patients [6]. For osteoporotic vertebral compression fractures that cannot be treated with conservative treatment, minimally invasive percutaneous vertebroplasty (PVP) and percutaneous kyphoplasty (PKP) are currently commonly used because it leads to small trauma and quick recovery. These two surgical methods have considerable applicability and acceptance in clinical settings [7, 8]. Owing to the improvement in social living conditions and the gradual extension of population life, more and more elderly patients are suffering from multiple vertebral compression fractures when they first visit hospitals. The vertebral body without fracture between the two fractured vertebral bodies is defined as a sandwich vertebral body. Some literature reports define it as the “Hamburg vertebral body.” In clinical practice, the sandwich vertebral can be fractured after PVP or PKP treatment of two fractured vertebrae. In this study, we aimed to investigate the risk of sandwich vertebral fracture during the treatment of osteoporotic vertebral compression fractures via PVP and PKP.

## Materials and methods

We performed a retrospective analytical study by including 61 patients with osteoporotic vertebral compression fractures who underwent PVP and PKP treatment at the Spinal Surgery Department of The Second Hospital of Liaocheng Affiliated with Shandong First Medical University from January 2019 to January 2022. These patients were divided into the following two groups by simple random sampling: group A (*N* = 30) received PVP treatment and group B (*N* = 31) received PKP treatment. PVP was performed through bilateral pedicle puncture, whereas PKP was performed through unilateral pedicle puncture. After surgery, both groups were treated with anti-osteoporosis drugs.

Selection criteria:

1. Osteoporotic vertebral compression fracture and non-traumatic fracture in elderly aged > 60 years.
2. Two or more thoracolumbar fractures including sandwich vertebral, as confirmed by MRI of fresh vertebral compression fractures; the posterior wall of the fractured vertebral body was complete without neurological symptoms.
3. All included cases showed obvious chest, waist, and back pain, with a VAS score > 5.

Exclusion criteria.

1. Pathological fractures such as vertebral tumors and vertebral hemangiomas.
2. Patients with a skin infection or abnormal coagulation function in the surgical puncture area.
3. Old compression fracture of the vertebral body, incomplete posterior wall of the vertebral body, and neurological symptoms.

### Surgical techniques

#### PVP surgical procedures in Group A

The patient was lying on the operating bed, with soft cushions placed in front of the chest and on both sides of the iliac spine to reduce abdominal pressure. A grid positioning plate was placed approximately at the position of the fracture vertebral body. A C-arm X-ray machine was used to perform fluoroscopy and locate the target fractured vertebral body. The operating table was adjusted to position the fractured vertebral body in a standard anteroposterior lateral position. The surface projection of the pedicle on both sides of the fractured vertebral body was marked. Routine disinfection and sheet laying was performed. Local anesthesia with 1% lidocaine was administered at 2–2.5 cm outside the projection of the vertebral pedicle body surface. The anesthesia was infiltrated layer by layer under C-arm fluoroscopy to the bone surface around the zygapophysial joint. Then, a 0.5 cm long incision was made using a sharp knife at the site of local anesthesia. A puncture needle was inserted into the outer edge of the articular process at both sides of the incision. After the C-arm fluoroscopy confirmed that the puncture point was in a good position, the puncture needle was rotated in the middle position of the vertebral pedicle when the fluoroscopy was in the right position. The C-arm was adjusted to the lateral position, and the fluoroscopy showed that the puncture needle was located at the half of the vertebral pedicle. C-arm fluoroscopy lateral view showed that the puncture needle was located at the posterior edge of the vertebral body, which indicated good internal and external inclination and head and tail inclination directions. The puncture needle continued to rotate into the vertebral body. Lateral fluoroscopy was used to locate the puncture needle at the front of 1/4th of the vertebral body. After the puncture needle position was appropriate, C-arm fluoroscopy was performed while injecting bone cement. The amount of bone cement in the thoracic spine was approximately 2–4 mL, and the amount of bone cement in the lumbar spine was approximately 4–6 mL. The puncture needle was extracted, and the surgical incision was covered to complete the surgery.

#### PKP surgical procedures in Group B

Position and puncture methods were the same as that for PVP surgery. A guide wire was inserted into the needle tube and the inner core was removed. After removing the cannula, the expansion catheter and the working cannula were installed. The expansion catheter and guide wire were removed. The fine drill was pushed to the front edge of the vertebral body. The guide needle was inserted and a cannula was placed along the guide needle. An expandable spherical airbag was inserted along the sleeve, which was inflated to expand. The airbag terminated at the edge of any cortex of the vertebral body, and the gas from the airbag was released and removed to establish a bone cavity in the vertebral body. Then, bone cement was injected into the cavity. After the hardening of the bone cement, the puncture needle was removed. The surgical incision was covered with sterile excipients and the surgery was completed.

## Results

**Fig. 1:**
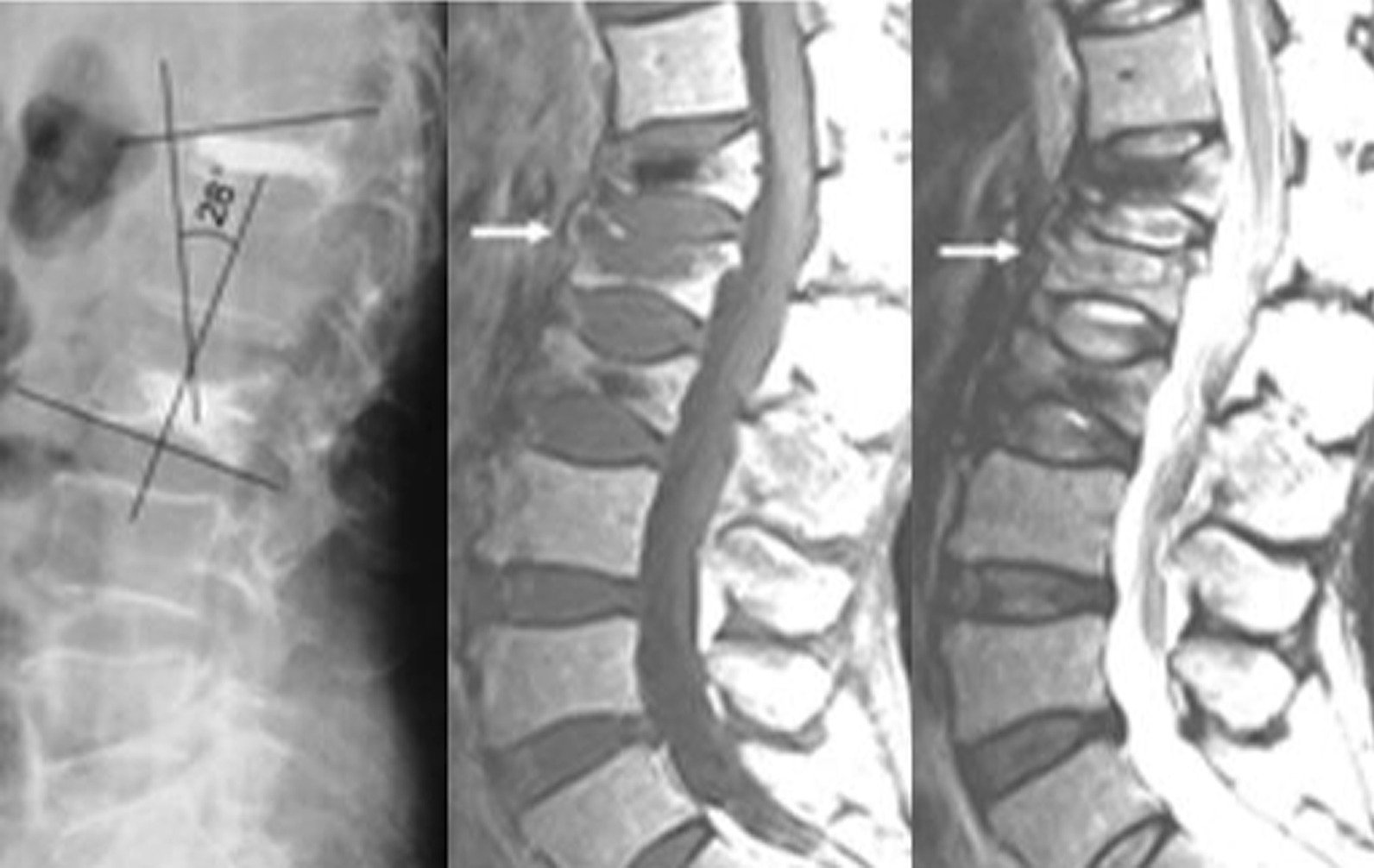
Cobb angle after PVP surgery

**Fig. 2:**
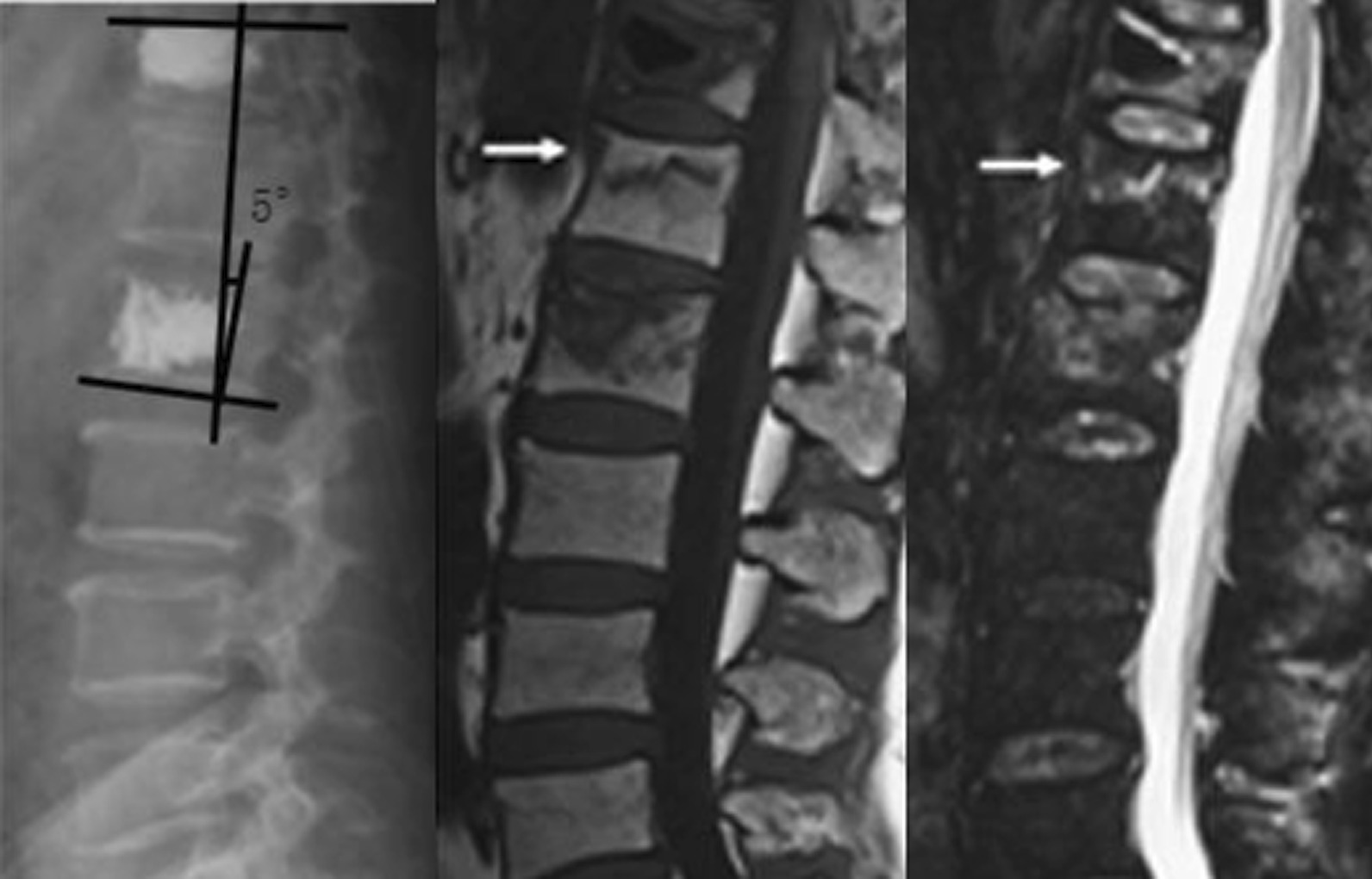
Cobb angle after PKP surgery

In this study, the patients were divided into two groups, namely A and B, based on the treatment that they underwent. Patients in group A underwent PVP, which included 30 patients. Patients in group B underwent PKP, which included 31 patients. The follow-up period was 1 year. Statistical analysis was performed using SPSS 26.0. The general characteristics of the patients in the two included age, gender, height, and weight. Age, height, and weight were compared using two independent sample *t*-tests, with *P* > 0.05, whereas gender was compared using a Chi-square test with *P* > 0.05. No statistically significant difference was found in the general characteristics of the two groups of patients (Table 1). Two independent sample *t*-tests were performed to compare surgical time, fluoroscopy frequency, VAS score, bone cement volume, and Cobb angle between the two groups of patients. The comparison of surgical time, fluoroscopy frequency, and VAS score between the two groups showed no statistically significant difference (*P* > 0.05). Conversely, the comparison of the amount of bone cement and Cobb angle between the two groups (Figs. 1, 2) showed a statistically significant difference (*P* < 0.05), with Group A being greater than Group B (Table 2). The leakage rate of bone cement intervertebral space in Group A was 20% (6/30), which was higher than that in Group B (3.23%, 1/31). Following up for one year, the incidence rate of vertebral fractures in Group A (36.67%, 11/30) was significantly higher than that in Group B (12.90%, 4/31). The leakage rate of bone cement intervertebral space and the incidence rate of vertebral fractures in the two groups were compared using the chi-square test (Significance at *P* < 0.05). The comparison between the two groups was statistically significant. The leakage rate of bone cement intervertebral space and the incidence rate of vertebral fractures in group A were both higher than those in group B (Table 2).

**Table 1:** General characteristics of the study cases

	PVP	PKP	*P*
Cases	30	31	
Age	78.90 ± 5.28	78.45 ± 5.32	0.78
Man/Female	8/22	8/23	0.96
Height(cm)	163.50 ± 7.41	163.45 ± 7.40	0.99
Weight (kg)	61.33 ± 11.11	62.51 ± 11.69	0.74

*P* < 0.05 was considered to indicate statistical significance

**Table 2:** Comparison between the two study groups

	PVP	PKP	*P*
Surgical time (min)	56.92 ± 5.04	57.58 ± 4.04	0.64
Fluoroscopy frequency	98.53 ± 6.64	97.29 ± 4.16	0.41
VAS scores	2.03 ± 0.77	1.81 ± 0.79	0.36
Amount of bone cement(mL)	9.97 ± 0.62	7.79 ± 0.48	< 0.001
Cobb angle	20.20 ± 1.81	8.13 ± 1.67	< 0.001
Leakage rate of bone cement			
Intervertebral space	6/30	1/31	0.03
Sandwich vertebral fracture	11/30	4/31	0.02

*P* < 0.05 was considered to indicate statistical significance

## Discussion

Osteoporotic vertebral compression fracture, the most common complication of osteoporosis, mostly occurs in the thoracic and lumbar vertebrae in older patients. It causes pain at the fracture site, loss of vertebral height, and later kyphosis. The main symptoms are pain and limited mobility at the fracture site [9], often manifested as increased pain when spine position changes: severe pain when lying in bed, turning over, and from bed to sitting up, seriously affecting the quality of life, and leading to death in severe cases.

Conservative treatment strategies have been used to treat osteoporotic vertebral compression fractures. Informal conservative treatment cannot relieve the pain symptoms but instead, can aggravate them. Long-term activity keeps the fractured vertebral body in a continuous compression state, and the slightly displaced fractured vertebral body continuously stimulates nerves around the vertebral body. Thoracic and lumbar fractures exhibit the highest compressive stress and subsequently develop deformities. These fractures mostly cause persistent back pain after the fracture. Osteoporotic thoracic compression fractures often present pain symptoms along the intercostal nerve: precordial area, lower xiphoid process, epigastrium, and lower abdomen, which is why some patients are often first diagnosed at the department of gastroenterology. Therefore, preventing deformity progression and correcting the existing deformity is crucial [10], for which they need to be diagnosed early so that reasonable surgical methods can be suggested to the patients.

Vertebral augmentation surgery, including PVP and PKP, is the widely used surgical treatment method. PVP and PKP can rapidly alleviate pain and restore a patient’s motor ability, mainly because bone cement eliminates the abnormal micromovement of the fractured vertebral trabecula and increases vertebral strength. In a randomized trial and meta-analysis, PVP and PKP showed better clinical results for patients with osteoporotic vertebral compression fractures than did conservative treatment strategies [11, 12]. Vertebral augmentation surgery is widely used in clinical cases owing to its advantages such as simple operation, minimal trauma, significant efficacy, and rapid recovery [13].

Owing to the widespread application of vertebral augmentation surgery, a standard surgical method for treating osteoporotic vertebral compressibility fractures in clinical practice, Clinical doctors can manage associated complications efficiently. Pulmonary embolism, spinal canal leakage, and postoperative infection have been consistently reported by many scholars. However, controversy exists about whether adjacent vertebral fractures are considered complications and the natural progression of osteoporosis. Recent studies suggest that vertebral augmentation surgery is associated with an increased risk of new fractures [14–16].

Many studies have compared the probability of fractures in interlayered vertebral bodies with adjacent and non-adjacent vertebral bodies after bone cement reinforcement; however, no study is available on the risk of fractures in interlayered vertebral bodies after PVP and PKP.

In this study, patients were divided into Groups A and B. Patients in Group A were treated with PVP, whereas those in Group B were treated with PKP to explore the risk of PVP and PKP for osteoporotic vertebral compression fractures on the occurrence of interbody fractures. The general characteristics of patients in the two groups, including age, gender, height, and weight, were statistically analyzed and the results showed no statistically significant difference in the general characteristics of the two groups (*P* > 0.05). The surgical time, fluoroscopy frequency, and postoperative VAS score of the two groups showed no statistically significant difference (*P* > 0.05). Conversely, the amount of bone cement, Cobb angle, the leakage rate of the intervertebral space, and the fracture rate of the sandwich vertebral body showed statistically significant differences between the two groups (*P* < 0.05).

During PVP surgery, we performed a bilateral pedicle puncture. The amount of bone cement in the vertebral body was higher than that observed during PKP. The vertebral bodies at both ends of the interbody were filled with more bone cement. Researchers found that when bone cement was injected into vertebral bodies with compression fractures, the stress of adjacent vertebral bodies increased significantly, causing adjacent vertebral fractures [17, 18].

The diffusion area of bone cement in the vertebral body observed during PVP was larger than that observed during PKP; owing to the higher amount of bone cement in PVP, bone cement can be easily distributed to the upper and lower endplates of the vertebral body during PVP. Studies have confirmed that the strength and hardness of bone cement distributed in the upper and lower endplates are greater than those of undistributed bone cement in the vertebral body [19, 20]. Therefore, the strength and hardness of the vertebral body during PVP are greater, and the mechanical conductivity of the sandwich vertebral body is greater, making it more prone to fractures. Theoretically, bone cement might increase the pressure on the adjacent disk, resulting in the deformation of the adjacent endplate, causing fractures in the endplate and nearby cancellous bone. Thus, stress and strain changes can further exacerbate, ultimately leading to adjacent vertebral fractures [21]. In the present study, when the Cobb angle was large, the fracture rate of the dissected vertebral body was higher, and PVP was higher than PKP. When spinal kyphosis occurs, local biomechanics change, and a body’s center of gravity shifts forward, requiring greater back muscle strength to maintain spinal balance. When the interlayered vertebral body cannot withstand the increased stress, fractures occur. In the present study, the leakage rates of bone cement in the intervertebral space of the two groups were 20% and 3.23%, respectively. The leakage of intervertebral space may be through the endplate and hollow fissure of the fracture. When the bone cement leaks into the intervertebral space, the force of the intervertebral space is transmitted to the sandwich vertebral body during the spinal load movement, subjecting the sandwich vertebral body to “double” force, making it prone to fractures. Herein, after the one-year follow-up, the fracture rates of the sandwich vertebral body in the two groups were 36.67% and 12.90%, respectively. Rates in the PVP group were significantly higher than those in the PKP group. Wang M et al. have confirmed that intervertebral disc leakage of bone cement is a risk factor for subsequent vertebral fractures [22].

### Limitations

Drug therapy for osteoporosis is particularly important after vertebroplasty for osteoporotic vertebral fractures; however, we did not study the effect of antiosteoporosis drug therapy on sandwich vertebral fractures. Furthermore, the number of cases in both groups was relatively small and the follow-up time was also short. Therefore, studies with expanded sample sizes and a long-term follow-up should be performed to verify the present findings.

## Conclusions

When PVP and PKP are used to treat osteoporotic vertebral compression fractures, the sandwich vertebral body possess a risk of fracture. PVP leads to a greater relative risk than PKP, which may be related to the relatively larger amount of bone cement, the higher rate of bone cement leakage in the intervertebral space, and the larger Cobb angle.

## Data Availability

All data generated or analyzed during this study are included in this article.
